# Improving prediction accuracy in acute myeloid leukaemia: micro-environment, immune and metabolic models

**DOI:** 10.1038/s41375-021-01377-0

**Published:** 2021-08-07

**Authors:** Fang Hu, Yun Wang, Wei-da Wang, Robert Peter Gale, Bing-yi Wu, Yang Liang

**Affiliations:** 1 Department of Hematologic Oncology, State Key Laboratory of Oncology in South China, Collaborative Innovation Center for Cancer Medicine, Sun Yat-sen University Cancer Center, Guangzhou, PR China; 2 Haematology Research Centre, Department of Immunology and Inflammation, Imperial College London, London, UK

**Keywords:** Cancer, Risk factors

## Introduction

Predicting the fate of someone with acute myeloid leukaemia (AML) at diagnosis is challenging [1, 2]. We recently reviewed several of these complexities in achieving accurate and precise estimates of outcomes in LEUKAEMIA [3]. Initial prediction efforts focused on clinical and laboratory co-variates such as WBC, percentage or numbers of myeloblasts and histology [4]. Cytogenetics data were soon added [5]. Most recently, data from studies of mutation topography, typically detected by targeted or next-generation sequencing (NGS), were added often displacing prior predictive co-variates. For example, the 2017 European Leukemia Net (ELN) model includes only data on cytogenetics and mutation topography. Predictive models using the expression pattern of genes related to leukaemia cell stemness are also reported [6]. Also new is the use of data from measurable residual disease (MRD)-testing but these data are not applicable to predicting outcomes at diagnosis [7]. The most recent predictive models divide persons with AML into more than 15 cohorts with statistically different prognoses [8–10]. Is this a clinically manageable number of predictive cohorts and are there convincing data these classifications are improving outcomes of persons with AML? Data so far show only a modest impact, if any [11]. For example, data from the US Surveillance and End Results (SEER) dataset indicate only a 10% 5-year survival improvement since 1999 (https://seer.cancer.gov/statfacts/html/amyl.html).

Most prediction models have concordance statistics (C-statistics) of 0.65–0.80 indicating only fair accuracy [3]. Can we do better? Are we too focused solely on leukaemia cell biology whilst ignoring other potentially important mechanisms influencing the complex interaction between the leukaemia and the host such as the bone marrow micro-environment and host immune response. Also, are there important aspects of the leukaemia cell biology we are likewise ignoring such as metabolism? Put otherwise, are there latent co-variates, co-variates that might improve prediction accuracy?

Recent studies suggest data regarding the bone marrow micro-environment, immune system and leukaemia cell metabolism might improve prediction accuracy. These data are reported to independently predict outcomes such as complete remission rate, cumulative incidence of relapse, event-, relapse- and leukaemia-free survivals (EFS, RFS and LFS) and/or survival in multi-variable analyses. Moreover, these co-variates are reported to improve the accuracy of more widely-used models such as the 2017 ELN model. We discuss these models below.

### Micro-environment and immune-risk models

Considerable data indicate cells in the bone marrow microenvironment, including immune, endothelial and stromal cells, the composition of the extracellular matrix and soluble factors such as cytokines, hepatocyte growth factor, vascular endothelial growth factor and angiopoietins are important in leukaemia development and progression [12]. The impact of the immune system in AML is increasingly studied. For example, there are several reports of correlations between blood and bone marrow natural killer (NK)-cells and survival [13, 14]. Specific T-cell phenotypes are also reportedly associated with leukaemia prognosis. For example, some data indicate pre- or post-therapy blood concentrations of PD-1+CD8+ T-cells and pre-therapy blood CD28-CD57+CD8+ T-cells correlate with EFS and survival [13]. Another study reported a high proportion of blood eomesodermin (Eomes +) T-bet^low^ CD8^+^ T-cells correlates with fewer complete remissions (CR) and worse survival [15].

Zhang et al. reported frequencies of CD4+CD25+CD127^lo^ regulatory T-cells (Tregs) in blood and bone marrow were associated to poor prognosis [16]. Han et al. reported increased inducible T-cell co-stimulator ligand positive Treg frequency in bone marrow was an unfavourable prognostic marker [17]. Kong et al. reported T-cell immunoglobulin and immune receptor tyrosine-based inhibitory motif domain (TIGIT) expression on blood CD8^+^ T-cells is increased in persons with AML and correlates with induction chemotherapy failure and with posttransplant relapse [18]. Several studies report high expression of PD-1, PD-L1 or PD-L2 was associated with poor survival [19, 20]. Increased co-expression of PD-1/CTLA-4 or PD-L2/CTLA-4 correlated with poor survival. Co-expression of PD-1/PD-L1, PD-1/PD-L1/PD-L2, or PD-1/LAG-3 correlated with poor survival in subjects with FLT3, RUNX1 and/or TET2 mutations [20]. Stamm et al. reported high PVR and PVRL2 expression, as novel immune checkpoints, correlated with poor outcomes [21].

**Fig. 1:**
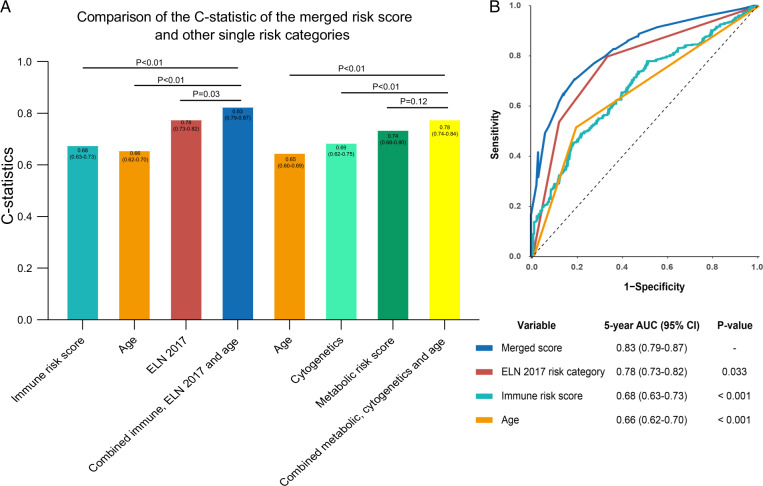
Comparison of C-statistics of the merged risk score and other single risk categories. **A** C-statistics were compared for prognostic co-variates and risk scores alone and combined. A higher C-statistic indicates better prediction accuracy. **B** Areas under the curve (AUC) of a receiver-operator characteristic (ROC) curve were compared for prognostic co-variates and risk scores alone or combined. The dashed line indicates no prediction accuracy. An increasing AUC indicates increasing prediction accuracy.

There are several predictive models of AML using data of immune cells identified by multi-parameter flow cytometry and/or NGS with bio-informatics. We reported an immune risk score derived from public datasets from Gene Expression Omnibus where we estimated proportions of immune cells in bone marrow samples using CIBERSORTx [22]. Data of six types of immune cells were used to develop prediction models for EFS and survival in persons receiving intensive induction chemotherapy. Prediction value of the model was validated in several datasets. Concentrations of activated NK-cells had the strongest predictive weight. The C-statistics of the immune risk score was 0.68 (0.63–0.73). However, A model adding the immune risk score to the ELN risk category (C-statistics 0.78 (0.73, 0.82) and age (C-statistics 0.66 [0.62–0.70]) had a revised C-statistics of 0.83 [0.79, 0.87]. The upper boundary of the 95% confidence interval is a marked improvement. Figure 1 displays the progressive improvement in prediction accuracy by combining different predictive co-variates and scores. Similar data are reported by others. For example, Bruck et al. identified several immune cell types with phenotypes in bone marrow correlated with prognosis including M1-polarised macrophages, FOXP3+ helper T-cells, Tregs and CTLA4^−^LAG3^−^ helper T-cells [23]. Dong et al. constructed a survival prediction model for persons with cytogenetically normal AML based on expression of 9 immune-related genes with C-statistics of 0.79 [24]. Zhu et al. reported a prediction model composed of 6 immune-related genes with an C-statistics 0.72 [25]. Cytokine profiles and interactions have also been used to predict outcomes of AML therapy. For example, one study reported correlations between serum concentrations of FLT3-ligand and interleukin-6 with LFS and survivalL [26]. Also, tumour necrosis factor-α, serum soluble interleukin-2 receptor-α (sIL2RA) and IL-10 concentration are reported to independently predict survival in AML [27–29]. In conclusion, immune-based prognostic and prediction models often complement and/or improve current AML prediction models.

### Metabolic-risk models

Another approach to improving prediction accuracy in AML involves leukaemia cell metabolism. Mutations in genes with metabolically active gene products such as isocitrate dehydrogenase isoform-1 (IDH1) and IDH2 are associated with changes in cell metabolism and possibly leukaemia initiation [30, 31] Chen et al. identified 47 metabolites significantly altered in serum samples from 400 subjects with AML compared with controls by gas chromatography time-of-flight mass spectrometry-based metabolomics [32]. They identified six serum glucose metabolites, lactate, 2-oxoglutarate, pyruvate, 2-hydroygluterate, glycerol-3-phosphate and citrate, whose concentrations correlated with EFS and survival in subjects receiving induction chemotherapy and validated in another cohort. Zhou et al. reported increased plasma concentrations of lysine and taurine predict outcomes of persons with AML-M2 [33].

Serum metabolomic profiling has also been used to identify metabolites associated with outcomes of children with AML receiving chemotherapy [34]. Higher levels of pantothenic acid were associated with response to cytarabine and with worse RFS. We and others reported prognostic models based on metabolism-related gene expression data from public datasets [35, 36]. Both models were validated with C-statistics of 0.88 and 0.78. Wang et al. [36] combined their metabolic model with cytogenetics and age co-variates improving the C-statistics for survival prediction from 0.69 and 0.65 to 0.78, a significant improvement.

### Other predictive co-variates

Two recent studies in older persons with AML using predominately conventional subject-related co-variates reported C-statistics of 0.72–0.74 similar to the C-statistic of the 2017 ELN risk classification [37, 38]. Predictive value of epigenetic regulatory genes such as DNMT3A and global methylation state have also been evaluated [39–41]. For example, a genome-wide methylation score is reported to predict outcomes of persons with AML with a higher methylation-score associated with a lower rate of complete remission [42]. Some alternative splicing events are also reported predict AML outcomes with C-statistics of 0.96 but without external validation [43]. Adding data of splicing signature improved prediction accuracy to the 2017 ELN risk classification with C-statistics of about 0.75 and a leukaemia stemness score combine with splicing signature improved prediction accuracy to the 2017 ELN risk classification with C-statistics of about 0.72 [44]. To the extent infection correlates with risk of death during intensive induction chemotherapy studies of the gastro-intestinal microbiome can also improve predicting EFS and survival [45].

### Therapy

Accurate prediction can improve therapy decisions in persons with AML [3]. Increasingly, physicians are aware of the importance of prediction accuracy in choosing competing therapies such as intensive induction therapy with cytarabine and daunorubicin versus less intensive therapy with azacytidine and venetoclax. Some recent studies report benefits of metabolic interventions such as enasideinib and ivosidenib but these are unconfirmed in randomised controlled trials [46].

## Discussion

**Fig. 2:**
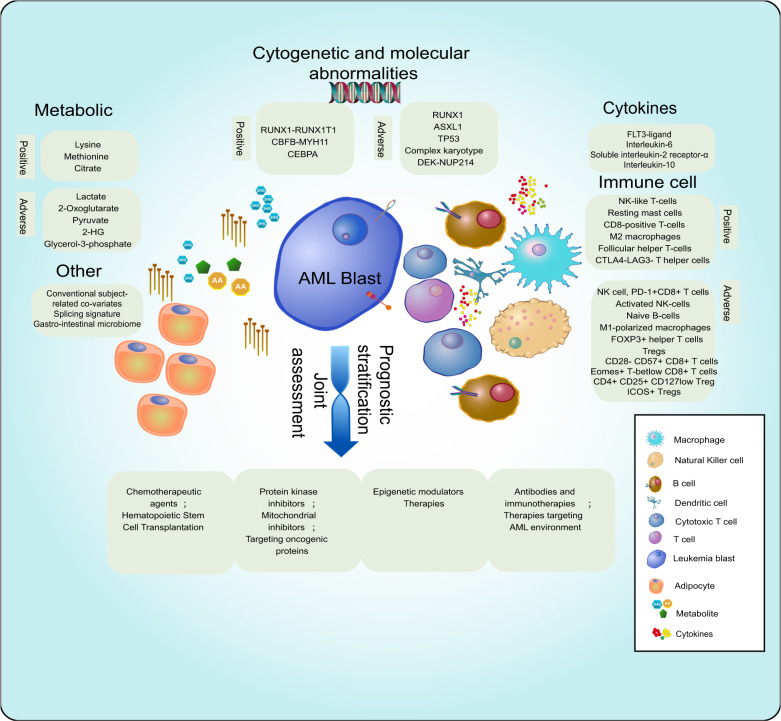
Immune, metabolic, cytogenetic and molecular co-variates correlated with therapy response and survival in persons with acute myeloid leukaemia receiving intensive induction chemotherapy. Figure show potential interactions.

Accurate prediction is fundamental to optimising AML therapy (Fig. 2). Data we discuss indicate micro-environment, immune and metabolism-related co-variates and others such as epigenetics and splicing gene profiles are independent outcome predictors in AML in multi-variable regression analyses. Adding these data to current prediction models increases accuracy including newer models incorporating mutation topography (Table 1).

**Table 1:** Predictive co-variates.

Immune model		
Ref	Favourable	Unfavourable
14	NK like T-cells	NK-cells
13	NI	PD-1^+^CD8^+^ T-cells, CD28^−^CD57^+^CD8^+^ T-cells
22	Resting mast cells, CD8^+^ T-cells, M2 macrophages, Follicular helper T-cells	Activated NK-cells, Naïve B-cells
23	CTLA4^−^LAG3^−^ T-helper cells	M1 polarised macrophages, FOXP3^+^ helper T-cells, Tregs
15	NI	Eomes^+^ T-bet^low^ CD8^+^ T-cells
16	NI	CD4^+^ CD25^+^ CD127^low^ Treg
17	NI	ICOS^+^ Tregs
20	NI	PD-1, PD-L1, PD-L2, Co-expression of PD-1/CTLA-4, Co-expression of PD-L2/CTLA-4
21	NI	PVR, PVRL2
Metabolic model		
32	Citrate	Lactate, 2-Oxoglutarate, Pyruvate, 2-Hydroxyglutarate, Glycerol-3-phosphate
33	Lysine, Methionine	NI
36	CYP2E1, HAAO, ITPKA, PAFAH1B2	ALDH2, DNMT3B, ENPP2, PHGDH, PSAT1
35	NI	PLA2G4A, HMOX2, AK1, SMPD3

None of current or newer prediction models we cite included data of MRD-testing at the end of therapy as an outcome predictor in model building. Whether current or new pre-therapy predictive models are better than results of post-therapy MRD-testing in predicting post-remission therapy outcomes is uncertain. Elsewhere we discuss the advantages and limitations of post-therapy MRD-testing as a predictor of subsequent outcomes in persons with AML [47]. Presently, MRD-testing data correlate strongly with outcomes but has high false-positive and -negative rates. Whether this limitation can be overcome is uncertain. Moreover, utility is limited to post-therapy setting rather than being useful at diagnosis.

Another issue is why and when do we want to determine prognosis or predict outcomes. Is it to identify the best initial therapy, say intensive therapy with cytarabine and daunorubicin, less intensive chemotherapy say with azacitidine with venetoclax, or a targeted therapy, say with enasideinib or ivosidenib? This decision is driven not only by the co-variates we identify but also by co-variates less often considered such as co-morbidities, access to medical care, expertise of the treating team, sophistication of supportive care, patient preference and economics. Or is our goal to predict what intervention should be given next. Obviously, this will be largely driven by outcome of the initial therapy and co-variates we cite above. For example, if the goal of an older person with substantial co-morbidities is to achieve the longest interval of high quality-of-life, achieving a complete remission may not be the appropriate therapy objective. In contrast, a young, otherwise healthy person may be willing to accept substantial adverse events for a chance, however small, of cure. In this instance a co-variate such as post-therapy MRD-testing may be the best predictive biomarker. What is important is that physicians and patients acknowledge our inaccuracy and imprecision in predicting outcomes of an intervention. What level of accuracy and precision is acceptable to drive a therapy decision is obviously subjective with no correct answer?

The new prediction models we discuss need optimisation and external validation in large datasets of uniformly-treated persons. To know if they are prognostic rather than predictive, they need to be tested in studies of diverse therapies. If validated they could be introduced into clinical practice and help with therapy decision-making.

In summary we show adding micro-environment-, immune- and metabolism-related and other co-variates improves prediction accuracy in newly-diagnosed persons with AML, predominately young people receiving intensive induction chemotherapy. Whether these co-variates are similarly useful in other therapy settings in unknown such as less intensive or targeted and immune. Because initial intensive therapy of AML is relatively uniform these co-variates are presently best regarded as predictive rather than prognostic. In appropriate therapy settings using prediction models which include micro-environment-, immune- and metabolism-related co-variates may be clinically useful. However, we need validation and optimisation in large prospective dataset of AML receiving diverse therapies such as cytarabine and daunorubicin versus azacitidine and venetoclax or enasideinib. When these are accomplished these new models be clinically-useful to predict outcomes and choose therapy(ies).

## References

[1] Khwaja A, Bjorkholm M, Gale RE, Levine RL, Jordan CT, Ehninger G (2016). Acute myeloid leukaemia. Nat Rev Dis Prim.

[2] Tachibana T, Kanda J, Ishizaki T, Najima Y, Tanaka M, Doki N (2019). Prognostic index for patients with relapsed or refractory acute myeloid leukemia who underwent hematopoietic cell transplantation: a KSGCT multicenter analysis. Leukemia.

[3] Estey E, Gale RP (2017). How good are we at predicting the fate of someone with acute myeloid leukaemia?. Leukemia.

[4] Bennett JM, Catovsky D, Daniel MT, Flandrin G, Galton DA, Gralnick HR (1976). Proposals for the classification of the acute leukaemias. French-American-British (FAB) co-operative group. Br J Haematol.

[5] Grimwade D, Walker H, Oliver F, Wheatley K, Harrison C, Harrison G (1998). The importance of diagnostic cytogenetics on outcome in AML: analysis of 1,612 patients entered into the MRC AML 10 trial. The Medical Research Council Adult and Children’s Leukaemia Working Parties. Blood.

[6] Ng SW, Mitchell A, Kennedy JA, Chen WC, McLeod J, Ibrahimova N (2016). A 17-gene stemness score for rapid determination of risk in acute leukaemia. Nature.

[7] Hourigan CS, Gale RP, Gormley NJ, Ossenkoppele GJ, Walter RB (2017). Measurable residual disease testing in acute myeloid leukaemia. Leukemia.

[8] Papaemmanuil E, Gerstung M, Bullinger L, Gaidzik VI, Paschka P, Roberts ND (2016). Genomic classification and prognosis in acute myeloid leukemia. N Engl J Med.

[9] Tazi Y, Arango JE, Zhou Y, et al. A unified classification and risk stratification algorithm to support clinical decisions in acute myeloid leukemia. Abstract #S133. EHA2021 Virtual Congress; 2021.

[10] Gerstung M, Papaemmanuil E, Martincorena I, Bullinger L, Gaidzik VI, Paschka P (2017). Precision oncology for acute myeloid leukemia using a knowledge bank approach. Nat Genet.

[11] Burd A, Levine RL, Ruppert AS, Mims AS, Borate U, Stein EM (2020). Precision medicine treatment in acute myeloid leukemia using prospective genomic profiling: feasibility and preliminary efficacy of the Beat AML Master Trial. Nat Med.

[12] Ayala F, Dewar R, Kieran M, Kalluri R (2009). Contribution of bone microenvironment to leukemogenesis and leukemia progression. Leukemia.

[13] Tang L, Wu J, Li CG, Jiang HW, Xu M, Du M (2020). Characterization of immune dysfunction and identification of prognostic immune-related risk factors in acute myeloid leukemia. Clin Cancer Res.

[14] Lion E, Willemen Y, Berneman ZN, Van Tendeloo VF, Smits EL (2012). Natural killer cell immune escape in acute myeloid leukemia. Leukemia.

[15] Jia B, Zhao C, Rakszawski KL, Claxton DF, Ehmann WC, Rybka WB (2019). Eomes(+)T-bet(low) CD8(+) T cells are functionally impaired and are associated with poor clinical outcome in patients with acute myeloid leukemia. Cancer Res.

[16] Shenghui Z, Yixiang H, Jianbo W, Kang Y, Laixi B, Yan Z (2011). Elevated frequencies of CD4(+) CD25(+) CD127lo regulatory T cells is associated to poor prognosis in patients with acute myeloid leukemia. Int J Cancer.

[17] Han Y, Dong Y, Yang Q, Xu W, Jiang S, Yu Z (2018). Acute myeloid leukemia cells express iCOS ligand to promote the expansion of regulatory T cells. Front Immunol.

[18] Kong Y, Zhu L, Schell TD, Zhang J, Claxton DF, Ehmann WC (2016). T-cell immunoglobulin and ITIM domain (TIGIT) associates with CD8+ T-cell exhaustion and poor clinical outcome in AML patients. Clin Cancer Res.

[19] Goltz D, Gevensleben H, Grunen S, Dietrich J, Kristiansen G, Landsberg J (2017). PD-L1 (CD274) promoter methylation predicts survival in patients with acute myeloid leukemia. Leukemia.

[20] Chen C, Liang C, Wang S, Chio CL, Zhang Y, Zeng C (2020). Expression patterns of immune checkpoints in acute myeloid leukemia. J Hematol Oncol.

[21] Stamm H, Klingler F, Grossjohann EM, Muschhammer J, Vettorazzi E, Heuser M (2018). Immune checkpoints PVR and PVRL2 are prognostic markers in AML and their blockade represents a new therapeutic option. Oncogene.

[22] Wang Y, Cai YY, Herold T, Nie RC, Zhang Y, Gale RP (2021). An immune risk score predicts survival of patients with acute myeloid leukemia receiving chemotherapy. Clin Cancer Res.

[23] Bruck O, Dufva O, Hohtari H, Blom S, Turkki R, Ilander M (2020). Immune profiles in acute myeloid leukemia bone marrow associate with patient age, T-cell receptor clonality, and survival. Blood Adv.

[24] Dong X, Zhang D, Zhang J, Chen X, Zhang Y, Zhang Y (2020). Immune prognostic risk score model in acute myeloid leukemia with normal karyotype. Oncol Lett.

[25] Zhu R, Tao H, Lin W, Tang L, Hu Y (2020). Identification of an immune-related gene signature based on immunogenomic landscape analysis to predict the prognosis of adult acute myeloid leukemia patients. Front Oncol.

[26] Peterlin P, Gaschet J, Guillaume T, Garnier A, Eveillard M, Le Bourgeois A (2021). A new cytokine-based dynamic stratification during induction is highly predictive of survivals in acute myeloid leukemia. Cancer Med.

[27] Kupsa T, Vanek J, Zak P, Jebavy L, Horacek JM (2020). Serum levels of selected cytokines and soluble adhesion molecules in acute myeloid leukemia: Soluble receptor for interleukin-2 predicts overall survival. Cytokine.

[28] Sanchez-Correa B, Bergua JM, Campos C, Gayoso I, Arcos MJ, Banas H (2013). Cytokine profiles in acute myeloid leukemia patients at diagnosis: survival is inversely correlated with IL-6 and directly correlated with IL-10 levels. Cytokine.

[29] Tsimberidou AM, Estey E, Wen S, Pierce S, Kantarjian H, Albitar M (2008). The prognostic significance of cytokine levels in newly diagnosed acute myeloid leukemia and high-risk myelodysplastic syndromes. Cancer.

[30] Medeiros BC, Fathi AT, DiNardo CD, Pollyea DA, Chan SM, Swords R (2017). Isocitrate dehydrogenase mutations in myeloid malignancies. Leukemia.

[31] Mylonas E, Janin M, Bawa O, Opolon P, David M, Quivoron C (2014). Isocitrate dehydrogenase (IDH)2 R140Q mutation induces myeloid and lymphoid neoplasms in mice. Leukemia.

[32] Chen WL, Wang JH, Zhao AH, Xu X, Wang YH, Chen TL (2014). A distinct glucose metabolism signature of acute myeloid leukemia with prognostic value. Blood.

[33] Zhou X, Zheng M, Wang Q, Aa J, Cao B, Li J (2020). Metabolomics analysis identifies lysine and taurine as candidate prognostic biomarkers for AML-M2 patients. Int J Hematol.

[34] Bradley Stockard PharmD, Huiyun Wu P, Joy D, Guingab P, Timothy J, Garrett P (2018). Metabolomics profiling reveals markers for chemosensitivity and clinical outcomes in pediatric AML patients. Blood.

[35] Zhang Y, Ma S, Wang M, Shi W, Hu Y (2020). Comprehensive analysis of prognostic markers for acute myeloid leukemia based on four metabolic genes. Front Oncol.

[36] Wang Y, Hu F, Li JY, Nie RC, Chen SL, Cai YY (2020). Systematic construction and validation of a metabolic risk model for prognostic prediction in acute myelogenous leukemia. Front Oncol.

[37] Liu CJ, Hong YC, Kuan AS, Yeh CM, Tsai CK, Liu YC (2020). The risk of early mortality in elderly patients with newly diagnosed acute myeloid leukemia. Cancer Med.

[38] Zhou F, Zhou F, Du M, Liu L, Guo T, Xia L (2019). Comprehensive prognostic scoring systems could improve the prognosis of adult acute myeloid leukemia patients. Int J Hematol.

[39] Jiang H, Ou Z, He Y, Yu M, Wu S, Li G (2020). DNA methylation markers in the diagnosis and prognosis of common leukemias. Signal Transduct Target Ther.

[40] Li Y, Xu Q, Lv N, Wang L, Zhao H, Wang X (2017). Clinical implications of genome-wide DNA methylation studies in acute myeloid leukemia. J Hematol Oncol.

[41] Ribeiro AF, Pratcorona M, Erpelinck-Verschueren C, Rockova V, Sanders M, Abbas S (2012). Mutant DNMT3A: a marker of poor prognosis in acute myeloid leukemia. Blood.

[42] Luskin MR, Gimotty PA, Smith C, Loren AW, Figueroa ME, Harrison J, et al. A clinical measure of DNA methylation predicts outcome in de novo acute myeloid leukemia. *JCI Insight*. 2016;1. 10.1172/jci.insight.87323 PMC4951094 27446991

[43] Chen SL, Dai YJ, Hu F, Wang Y, Li H, Liang Y (2020). Effects of alternative splicing events on acute myeloid leukemia. DNA Cell Biol.

[44] Anande G, Deshpande NP, Mareschal S, Batcha AMN, Hampton HR, Herold T (2020). RNA splicing alterations induce a cellular stress response associated with poor prognosis in acute myeloid leukemia. Clin Cancer Res.

[45] Galloway-Pena JR, Shi Y, Peterson CB, Sahasrabhojane P, Gopalakrishnan V, Brumlow CE (2020). Gut microbiome signatures are predictive of infectious risk following induction therapy for acute myeloid leukemia. Clin Infect Dis.

[46] Stein EM, DiNardo CD, Fathi AT, Mims AS, Pratz KW, Savona MR (2021). Ivosidenib or enasidenib combined with intensive chemotherapy in patients with newly diagnosed AML: a phase 1 study. Blood.

[47] Othus M, Wood BL, Stirewalt DL, Estey EH, Petersdorf SH, Appelbaum FR (2016). Effect of measurable (‘minimal’) residual disease (MRD) information on prediction of relapse and survival in adult acute myeloid leukemia. Leukemia.

